# Emotional regulation, economic predictors, and their influence on life satisfaction in Peruvian university students

**DOI:** 10.3389/fpsyg.2026.1892186

**Published:** 2026-07-17

**Authors:** Vilma Vilca-Pareja, Manuel Edmundo Hillpa-Zuñiga, María Elena Rojas-Zegarra, Victor Ritchar Yana-Calla, Angel Roland Ugarte-Concha

**Affiliations:** 1 Escuela de Posgrado, Estudios Generales, Universidad Católica de Santa María, Arequipa, Peru; 2 Faculty of Administration and Business, Universidad Tecnológica del Perú, Lima, Peru; 3 Escuela Profesional de Psicología de la Universidad Nacional de San Agustín de Arequipa, Arequipa, Peru; 4 Escuela Profesional de Administración de Empresas, Facultad de Ciencias Económico Administrativas, Universidad Católica de Santa María, Arequipa, Peru

**Keywords:** cognitive reappraisal, economic predictors, emotional regulation, expressive suppression, life satisfaction

## Abstract

**Introduction:**

This study investigates how emotional regulation and economic predictors influence life satisfaction. The mental health of university students is currently a global concern. Therefore, it is essential to identify protective factors that promote well-being in this population.

**Method:**

A total of 1,030 students participated in the study (624 women and 406 men; aged between 18 and 30 years) from two private universities in Arequipa, Peru (M = 19.9; SD = 2.2).

**Results:**

The findings showed that cognitive reappraisal was positively related to life satisfaction (*r* = 0.255) and economic predictors (*r* = 0.173). Expressive suppression showed negative relationships with life satisfaction (*r* = −0.095). Subsequently, a PLS SEM analysis was performed, where the exogenous variables such as emotional regulation and economic predictors explain 35.9% of the variance in life satisfaction (*R*^2^ = 0.359).

**Discussion:**

These findings suggest that interventions aimed at improving well-being should adopt a multidimensional approach, integrating both the strengthening of emotional regulation skills and economic well-being among university students.

## Introduction

Subjective well-being (SWB) has become one of the central constructs in contemporary psychology, particularly within the framework of positive psychology and behavioral sciences, as it integrates cognitive and affective components that reflect the overall evaluation individuals make of their own lives (Diener et al., 2018; Diener, 1984). Since the classical formulation proposed by Diener (1984), subjective well-being has been conceptualized as a structure composed of positive affect, negative affect, and life satisfaction, the latter being the cognitive component of the construct. In operational terms, life satisfaction is defined as the evaluative judgment individuals make regarding the extent to which their life conditions match their expectations and personal standards (Diener et al., 1985). This definition highlights the subjective and comparative nature of well-being, which depends not only on objective conditions but also on internal evaluative processes.

Over recent decades, research has advanced toward identifying the factors that explain interindividual differences in life satisfaction, emphasizing the importance of integrating psychological and economic variables into explanatory models of well-being (Ruggeri et al., 2020; Jebb et al., 2020). This approach recognizes that well-being is not solely the result of favorable external conditions but also depends on the cognitive and emotional processes through which individuals interpret and cope with their environment (Lazarus and Folkman, 1984), as well as on the subjective perception of their economic resources (Netemeyer et al., 2018; Fan and Henager, 2022).

From the process model of emotion regulation, regulatory strategies differ according to the moment at which they intervene in the generation of the emotional response. In particular, cognitive reappraisal constitutes an antecedent-focused strategy that allows individuals to reinterpret potentially stressful situations before the emotional response is fully generated, whereas expressive suppression involves inhibiting emotional expression once the response has been activated (Gross and John, 2003; Gross, 2015). Empirical evidence has consistently demonstrated that cognitive reappraisal is associated with higher levels of well-being, better psychological adjustment, and greater life satisfaction, whereas expressive suppression is linked to less adaptive outcomes, including higher stress and lower well-being (Gross, 2015; Hu et al., 2014; Cabello et al., 2025). Recent studies also confirm that emotion regulation plays a key role in psychological adaptation and in the subjective evaluation of life (Potoczny et al., 2025; Zhao et al., 2024). In parallel, the economic dimension has taken on a central role in explaining subjective well-being. Contemporary evidence suggests that the subjective perception of economic adequacy constitutes a more relevant predictor than objective income indicators (Netemeyer et al., 2018; Riitsalu et al., 2024). Along these lines, recent studies have shown that the economic dimension is positively associated with life satisfaction; conversely, financial stress is related to lower well-being and greater psychological distress (Ryu and Fan, 2023; Bialowolski et al., 2021). Likewise, empirical research has shown that financial satisfaction mediates the relationship between economic practices and subjective well-being (Atatsi et al., 2023), reinforcing the importance of this dimension in the overall evaluation of life.

In contexts of crisis and uncertainty, such as the COVID-19 pandemic, the relevance of economic factors has intensified, with evidence showing a significant impact of financial instability on mental health and life satisfaction (Robillard et al., 2020; Antipova, 2021). In this regard, recent studies have demonstrated that the perception of economic security and financial control acts as a key resource for psychological stability (Copur and Dogan, 2023) and may even moderate the impact of adverse events on well-being (Clench-Aas et al., 2021).

Within the Latin American context, and particularly in Peru, empirical evidence regarding the relationship between economic predictors and life satisfaction has shown consistent results. In this sense, the study conducted by Mamani-Benito et al. (2025) demonstrated that economic predictors positively influence life satisfaction, whereas financial stress has negative effects, confirming the relevance of subjective economic factors in explaining well-being among the Peruvian working population.

Despite the robustness of the international evidence, gaps remain in the simultaneous integration of emotional and economic variables into explanatory models of subjective well-being. Most studies have addressed these factors independently, which limits the understanding of their joint contribution. Recent research based on configurational approaches suggests that well-being emerges from the interaction of multiple conditions, including psychological, economic, and relational factors (Jalal-Ahamed, 2025), highlighting the need for integrative models.

Within this framework, the present study aims to: (a) establish the relationship between emotion regulation, economic predictors, and life satisfaction; and (b) analyze the predictive role of cognitive reappraisal, expressive suppression, and economic predictors on life satisfaction. The following hypotheses are proposed:

Hypothesis 1: Cognitive reappraisal will be positively associated with life satisfaction.

Hypothesis 2: Expressive suppression will be negatively associated with life satisfaction.

Hypothesis 3: Cognitive reappraisal, expressive suppression, and economic predictors will significantly predict life satisfaction.

Overall, this study seeks to generate empirical evidence integrating psychological and economic factors in the explanation of well-being within a specific sociocultural context, while offering relevant implications for the design of interventions aimed at promoting well-being from a multidimensional approach.

## Methods

### Participants

The study was conducted at two private universities in Arequipa, Perú. A total of 1,030 students participated in the study. A non-probabilistic purposive sampling method was used. Participants’ ages ranged from 18 to 30 years (M = 19.9, SD = 2.2); 60.6% were women and 39.4% were men. Inclusion criteria were being enrolled university students between 18 and 30 years of age and providing written informed consent. 72.6% (*n* = 748) of the participants only dedicate themselves to study and 27.4% (*n* = 282) work and study at the same time.

### Instruments

#### Emotion regulation questionnaire (ERQ)

This questionnaire was developed by Gross and John (2003). The Spanish adaptation of the scale developed by Cabello et al. (2013). In Peru, the instrument was validated by Gargurevich and Matos (2010), who confirmed the construct validity and reliability of the instrument, with Cronbach’s alpha coefficients above 0.70 for both dimensions. The instrument consists of two dimensions: (a) cognitive reappraisal, considered a strategy for regulating emotions; and (b) expressive suppression, which involves modulating the ongoing emotional response. The scale consists of 10 items: four items are related to expressive suppression and six items to cognitive reappraisal. It uses a 7-point Likert scale ranging from 1 (“strongly disagree”) to 7 (“strongly agree”). A representative item from this scale is: “When I am faced with a stressful situation, I try to think about it in a way that helps me stay calm.”

#### Economic predictors

Economic predictors include income, which enables individuals to satisfy basic needs such as housing, clothing, food, and education, as well as saving and borrowing capacity (Simonse et al., 2024). This construct is assessed through items designed to measure satisfaction with income and saving capacity, as well as the quality of material aspects of life, such as housing, clothing, food, and personal possessions. A representative item from this scale is: “What is your level of satisfaction regarding the equipment and information technologies you own, such as computers, laptops, cell phones, internet access, etc.?”

The instrument was designed by the authors and consists of 5 items (a unidimensional scale), using a 5-point Likert-type response scale ranging from 1 (“strongly disagree”) to 5 (“agree”).

To verify evidence of validity, the instrument was subjected to expert judgment, in which experts evaluated the coherence, relevance, and clarity of the items. Content validity was assessed using Aiken’s *V* coefficient to measure expert agreement, for this end, the suggested cut-off point of 0.70 (Napitupulu et al., 2018; Escurra, 1969) was considered; the values obtained for the 5 items were greater than 0.94 (items 2, 3 and 5 Aiken’s *V* = 0.94; items 1 and 4 Aiken’s *V* = 1.0). Likewise, a pilot study was conducted with a sample of 200 participants. Subsequently, a Confirmatory Factor Analysis (CFA) was performed, obtaining adequate fit indices (RMSEA = 0.04; CFI = 0.93; and TLI = 0.95), using an MLR estimator to assess internal structure validity. Finally, the reliability of the instrument was evaluated through Cronbach’s alpha and McDonald’s omega coefficients (*α* = 0.825 and *ω* = 0.830, indicating that the instrument is reliable for measuring economic predictors).

#### Satisfaction with Life Scale

The Satisfaction with Life Scale assesses the cognitive component of subjective well-being. It was originally developed by Diener et al. (1985) and later adapted to the Spanish-speaking context by Atienza et al. (2000), The scale consists of five items answered using a 7-point Likert-type scale ranging from 1 (“strongly disagree”) to 7 (“strongly agree”). In the Peruvian context, Calderón-De la Cruz et al. (2018) analyzed the psychometric properties of the brief version, confirming the internal structure of the instrument through Confirmatory Factor Analysis. Likewise, internal consistency was estimated using omega (*ω* = 0.863) and *H* (0.920) coefficients, showing adequate levels of reliability. A representative item from this scale is: So far, I have gotten the important things I want in life.”

#### Procedure

This study was approved by the Ethics Committee of Universidad Católica de Santa María de Arequipa (278-2025) prior to its implementation. The instruments were administered virtually through the TEAMS platform. Data were coded in Excel 360, and a database was subsequently created for export to the R software environment and the RStudio application, where the corresponding statistical analyses were conducted. The Partial Least Squares Structural Equation Modeling (PLS-SEM) analysis was conducted using the SEMinR package (version 4.5.3), an open-source software tool designed for variance-based structural equation modeling.

## Results

### Descriptive statistics of the items

Descriptive statistics were calculated using the mean, standard deviation, skewness, and kurtosis.

Table 1 presents the descriptive statistics for the variables emotion regulation, economic predictors, and life satisfaction. Relatively high scores were observed for cognitive reappraisal (M = 29.5, SD = 6.1) and intermediate levels for expressive suppression (M = 17.0, SD = 4.8), suggesting a moderate use of these emotional strategies among participants. Economic predictors showed a mean of 16.9 (SD = 3.8), whereas life satisfaction presented a mean of 22.6 (SD = 5.8), reflecting moderate levels of satisfaction with life. The observed standard deviations indicate adequate variability in responses. On the other hand, the skewness (*g*_1_) and kurtosis (*g*_2_) coefficients were within acceptable ranges (−1 to +1), allowing the assumption of approximate normality of the distributions (George and Mallery, 2016). A slight negative skewness was observed across all variables, with relatively symmetrical distributions overall, suggesting that the scores were evenly distributed within the sample and met the assumptions necessary for subsequent parametric statistical analyses.

**Table 1:** Descriptive and shape statistics for the study variables.

Variable	Min	Max	M	SD	*g*_1_	*g*_2_
1. Revaluation cognitive	6	42	29.5	6.1	−0.26	0.36
2. Suppression expressive	4	28	17.0	4.8	−0.15	0.12
3. Economic predictors	5	25	16.9	3.8	−0.44	0.07
4. Life satisfaction	5	35	22.6	5.8	−0.39	−0.12

Min, minimum value; Max, maximum value; M, mean; SD, standard deviation; *g*_1_ skewness; g_2_, kurtosis.

Table 2 presents the Pearson correlation coefficients among the study variables, together with the internal consistency indices of the scales used. Overall, the results show a consistent pattern of significant relationships among the analyzed variables. First, cognitive reappraisal was positively and statistically significantly associated with expressive suppression (*r* = 0.226, *p* < 0.001), economic predictors (*r* = 0.173, *p* < 0.001), and life satisfaction (*r* = 0.255, *p* < 0.001), suggesting that greater use of adaptive emotion regulation strategies is related to a more favorable economic perception and higher levels of life satisfaction. In contrast, expressive suppression showed negative and significant correlations with economic predictors (*r* = −0.039, *p* < 0.05) and life satisfaction (*r* = −0.095, *p* < 0.01), indicating that the use of this less adaptive regulatory strategy is associated with lower evaluations of life satisfaction, in line with previous literature on emotion regulation (Gross and John, 2003). Likewise, economic predictors showed a moderate positive correlation with life satisfaction (r = 0.517, *p* < 0.001), reinforcing empirical evidence highlighting the relevance of economic predictors in the overall evaluation of life satisfaction (Diener et al., 2018). Finally, Cronbach’s alpha coefficients ranged from 0.71 to 0.85, exceeding the recommended threshold of 0.70, thereby demonstrating adequate internal consistency and reliability of the scales used and supporting the validity of the statistical analyses conducted (Tavakol and Dennick, 2011).

**Table 2:** Pearson correlations among the study variables.

Variable	*α*	1	2	3	4
1. Revaluation cognitive	0.773	–			
2. Suppression expressive	0.711	0.226^**^	–		
3. Economic predictors	0.852	0.173^**^	−0.039^*^	–	
4. Life satisfaction	0.844	0.255^**^	−0.095^**^	0.517^**^	–

M, mean; SD, standard deviation; *α*, Cronbach’s alpha.

**p < 0.01; **p < 0.05.

For a better understanding and validity of the model, the modeling of structural equations by minimum squares (PLS-SEM). This study analyzes latent variables, measured through multiple items, like simultaneous predictive relations between exogenous variables (cognitive reappraisal, expressive suppression, and economic predictors) and an endogenous variable (life satisfaction). Furthermore, PLS-SEM allows to evaluate in a joint way the measure and structural model (Hair et al., 2021). In order to obtain a valid model, items 1 (cognitive reappraisal) and 9 (suppression) of the emotional regulation, since their weights did not achieve 0.40 value (Hair et al., 2021). The results for the PLS SEM can be seen in Table 3. First, in the analysis of the measurement, results showed adequate reliability values, since the alfa values varied between 0.704 and 0.852, while the composite reliability showed values between 0.829 and 0.894, improving confidentiality; likewise, convergent validity was adequate, given that the AVE values are between 0.500 and 0.630 (Hair et al., 2021; Henseler et al., 2015). Regarding the discriminant validity of the measurement model using the HTMT criterion, its values are between 0.080 and 0.620, all below the cutoff point of 0.85, which indicates that the constructs are different (Henseler et al., 2015). Similarly, no collinearity problems were identified among the predictors, given that the VIF values were low: cognitive reappraisal = 1.071, expressive suppression = 1.041 and economic predictors = 1.041 (Hair et al., 2021).

**Table 3:** Assessment of the PLS SEM measurement model.

Variables	CR	AVE	HTMT				Collinearity
			1	2	3	4	VIF
1. Cognitive reassessment	0.829	0.500	_				1.071
2. Expressive suppression	0.829	0.620	0.261	_			1.041
3. Economic predictors	0.894	0.630	0.223	0.080	_		1.041
4. Life satisfaction	0.888	0.620	0.353	0.170	0.620	_	_

CR, composite reliability; AVE, mean variance extracted; HTMT, heterotrait–monotrait ratio; VIF, variance inflation factors.

**Figure 1:**
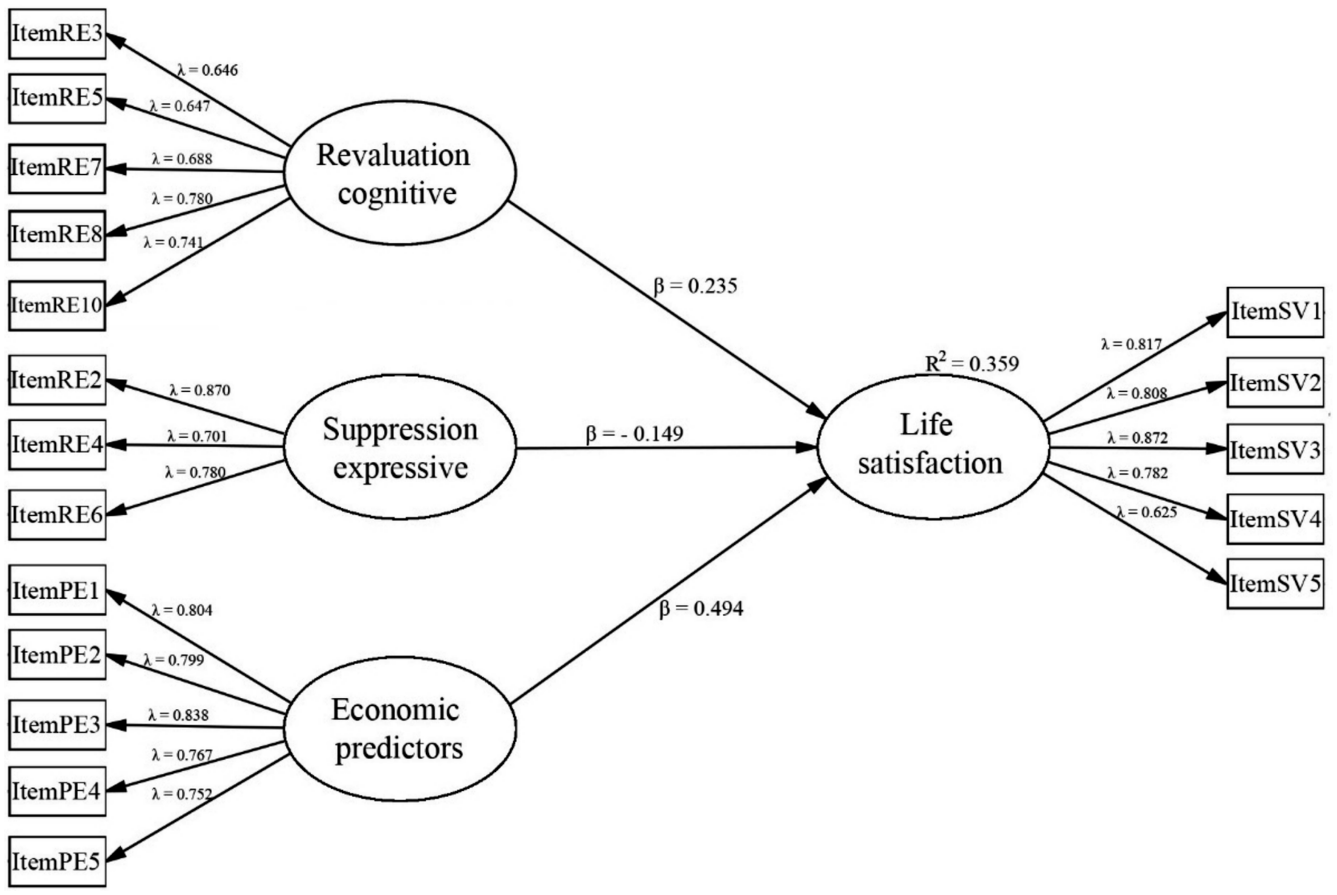
PLS SEM structural model.

Regarding the structural model, exogenous variables explain 35.9% of the variance in life satisfaction (*R*^2^ = 0.359). The variable that has the greatest effect is economic predictors, *β* = 0.494, followed by cognitive reappraisal, *β* = 0.235. However, expressive suppression had an inverse effect on life satisfaction, *β* = −0.149. These results show that life satisfaction is strongly associated with economic predictors and cognitive reappraisal, while expressive suppression is weakly and negatively associated with life satisfaction. Likewise, the external (factorial) loads of the indicators were located between *λ* = 0.625 and *λ* = 0.872, which shows an acceptable contribution of the items to their respective constructs (Hair et al., 2021). These results are presented in Figure 1.

## Discussion

The present study aimed to analyze the role of emotion regulation and economic predictors in explaining life satisfaction. Overall, the results support the multidimensional nature of subjective well-being, demonstrating the interaction between psychological and economic factors, which is consistent with contemporary approaches that conceptualize well-being as a complex and integrated phenomenon (Ruggeri et al., 2020; Jebb et al., 2020).

Regarding the first hypothesis, the results confirm that cognitive reappraisal is positively and significantly associated with life satisfaction, both at the correlational level and within the structural equations model. This finding is consistent with previous literature identifying cognitive reappraisal as a key adaptive emotion regulation strategy. Recent studies have shown that this strategy enables individuals to reinterpret adverse situations, reducing their negative emotional impact and facilitating more positive evaluations of life (Potoczny et al., 2025; Zhao et al., 2024). From an explanatory perspective, cognitive reappraisal promotes more efficient coping processes, fostering emotional stability and a greater sense of control over experiences, which translates into higher levels of life satisfaction, in line with recent empirical evidence on emotion regulation and well-being (Potoczny et al., 2025; Zhao et al., 2024). Nevertheless, the magnitude of the effect suggests that although this strategy is relevant, its influence is smaller in comparison with broader contextual factors, which is consistent with integrative models of subjective well-being (Jebb et al., 2020).

With regard to the second hypothesis, the results support the negative relationship between expressive suppression and life satisfaction. This pattern is consistent with research indicating that suppression involves greater cognitive and emotional costs, negatively affecting psychological well-being (Zhao et al., 2024). Inhibiting emotional expression may generate discrepancies between internal experience and observable behavior, reducing emotional authenticity and potentially affecting interpersonal relationships, thereby indirectly impacting the overall evaluation of life. This has been documented in recent studies on emotional intelligence and well-being (Zhao et al., 2024). However, the relatively small effect size found suggests that although this strategy is maladaptive, it has a limited impact on overall well-being compared to other more structural determinants, which is consistent with evidence emphasizing the predominant role of economic factors (Jebb et al., 2020).

Regarding the third hypothesis, the results clearly demonstrate that economic predictors constitute the factor with the greatest explanatory weight in life satisfaction. The strong association observed, both in correlational terms, and in the structural equations model, confirms that economic predictors are determinants of well-being. This finding is consistent with studies highlighting the role of financial well-being as a robust predictor of life satisfaction (Netemeyer et al., 2018; Fan and Henager, 2022). Likewise, recent studies have shown that financial satisfaction acts as a mediator between economic practices and overall well-being (Atatsi et al., 2023), reinforcing the importance of this dimension. In contexts of economic uncertainty, such as those resulting from recent crises, this effect may intensify, given that financial stress and economic insecurity are associated with lower levels of psychological well-being (Ryu and Fan, 2023; Bialowolski et al., 2021). Similarly, evidence from Latin American contexts has shown that subjective financial well-being constitutes a key element in the evaluation of quality of life (Estela-Delgado et al., 2023). From a theoretical perspective, these results may be interpreted through structural models of financial well-being, which propose that the perception of economic security directly influences the overall evaluation of life and the ability to cope with stressful events (Riitsalu et al., 2024), thereby supporting the central role of economic factors in explaining subjective well-being (Netemeyer et al., 2018).

Taken together, the model explained 35.9% of the variance in life satisfaction. This result is consistent with previous studies integrating psychological and economic variables into explanatory models of well-being, which typically report similar levels of explained variance (Park et al., 2019; Chilver et al., 2023). This finding reinforces the idea that subjective well-being is a complex and multifactorial construct influenced by multiple interrelated dimensions, as suggested by contemporary models of well-being (Ruggeri et al., 2020). Likewise, the model shows that economic factors have greater explanatory weight compared to emotion regulation variables, suggesting that the impact of psychological processes may be conditioned by the structural context in which they develop. In this regard, recent research has indicated that variables such as financial stress may moderate the influence of psychological factors on well-being (Kim, 2024; Nasir et al., 2025), supporting the interaction between individual and contextual factors.

The results support an integrative perspective of subjective well-being, in which emotion regulation and economic predictors operate in a complementary manner. In line with recent configurational approaches, well-being does not depend on a single factor, but rather on the interaction of multiple conditions, including psychological, economic, and contextual dimensions (Jalal-Ahamed, 2025), reinforcing the need for comprehensive explanatory models of well-being. In applied terms, these findings suggest that interventions aimed at improving life satisfaction should adopt a multidimensional approach, integrating both the strengthening of emotion regulation skills and strategies aimed at improving perceptions of economic security and financial control, especially in contexts characterized by economic vulnerability, in accordance with recent evidence on financial well-being (Fan and Henager, 2022).

Regarding the contributions of the study, this research provides new empirical evidence for the Peruvian context by integrating, in the same explanatory model, variables traditionally analyzed independently: emotional regulation, economic predictors and satisfaction with life. Likewise, it expands the literature on subjective well-being by demonstrating that the perception of financial security and emotional competencies act in a complementary way in the global assessment of well-being, providing evidence from an underrepresented in international studies Latin American university population. However, the results must be interpreted considering a few limitations. The cross-sectional design prevents establishing causal relationships between variables, while the use of self-report measures may be influenced by social desirability and subjective perception bias. Furthermore, the sample is limited to Peruvian university students, which limits the generalization of the findings to other population groups. Future research could employ longitudinal designs, incorporate objective indicators of economic status, and explore mediational or moderating models that achieve a more comprehensive understanding of the mechanisms by which emotional and financial resources influence life satisfaction.

## Data Availability

The raw data supporting the conclusions of this article will be made available by the authors, without undue reservation.
